# HHV-8 reduces dendritic cell migration through down-regulation of cell-surface CCR6 and CCR7 and cytoskeleton reorganization

**DOI:** 10.1186/1743-422X-9-92

**Published:** 2012-05-14

**Authors:** Mara Cirone, Valeria Conte, Antonella Farina, Sandro Valia, Pankaj Trivedi, Marisa Granato, Roberta Santarelli, Luigi Frati, Alberto Faggioni

**Affiliations:** 1 Istituto Pasteur-Fondazione Cenci Bolognetti, Department of Experimental Medicine, La Sapienza University, Viale Regina Elena 324, 00161, Rome, Italy

**Keywords:** Chemokines, Chemokine receptors, HHV-8, Dendritic cells.

## Abstract

**Background:**

For an efficient immune response against viral infection, dendritic cells (DCs) must express a coordinate repertoire of receptors that allow their recruitment to the sites of inflammation and subsequently to the secondary lymphoid organs in response to chemokine gradients.

Several pathogens are able to subvert the chemokine receptor expression and alter the migration properties of DCs as strategy to escape from the immune control.

**Findings:**

Here we report the inhibitory effect of Human Herpesvirus 8 (HHV-8) on the migratory behavior of immature and mature DCs. We found that the virus altered the DC chemokine receptor expression and chemokine induced migration. Moreover HHV-8 was also able to interfere with basal motility of DCs by inducing cytoskeleton modifications.

**Conclusion:**

Based on our findings, we suggest that HHV-8 is able to subvert the DC migration capacity and this represents an additional mechanism which interferes with their immune-functions.

## Findings

Dendritic cells (DCs) are critical for initiating an adaptive immune response against microbial and tumor antigens [1]. Their function is strictly linked to their migration properties along chemokine gradients and other chemotactic factors [2]. Immature DCs (iDCs) and their precursors are recruited to the infection and inflammatory sites in response to inflammatory chemokines such as MIP1 alfa and beta, CCL5/Rantes and CCL20 which bind to the chemokine receptors CCR1, CCR5, CCR2 and CCR6 respectively [3-5].In the peripheral tissues, DCs can initiate the antigen uptake and processing which, together with the exposure to inflammatory signals, results in DC maturation. This process is followed by switch of the chemokines receptor repertoire, e.g. downregulation of CCR5 and upregulation of CCR7, which drives their migration to secondary lymphoid organs mainly in response to the CCL19 chemokine [6]. To evade immune control, several viruses have been shown to interfere with the migratory capacity of DCs, as well as with their differentiation and maturation process. Vaccinia virus impairs migration and receptor switch of infected and bystander DCs [7], Human Respiratory Syncitial virus [8], Herpes Simplex virus [9] and Cytomegalovirus alter the chemokine-mediated migration by downregulating CCR1, CCR5 [10] and CCR7 [11] chemokine receptors on immature and mature DCs. Human Herpesvirus 8 (HHV-8), an oncogenic virus associated to Kaposi’s Sarcoma and Primary Effusion Lymphoma [12] has been previously shown to interfere with DC function [13] and impair the in vitro monocyte differentiation into DCs [14]. Here we asked if HHV-8 is also able to alter the pattern of chemokine receptor expression of monocyte-derived DCs and interfere with their migratory capacity.

**Figure 1:**
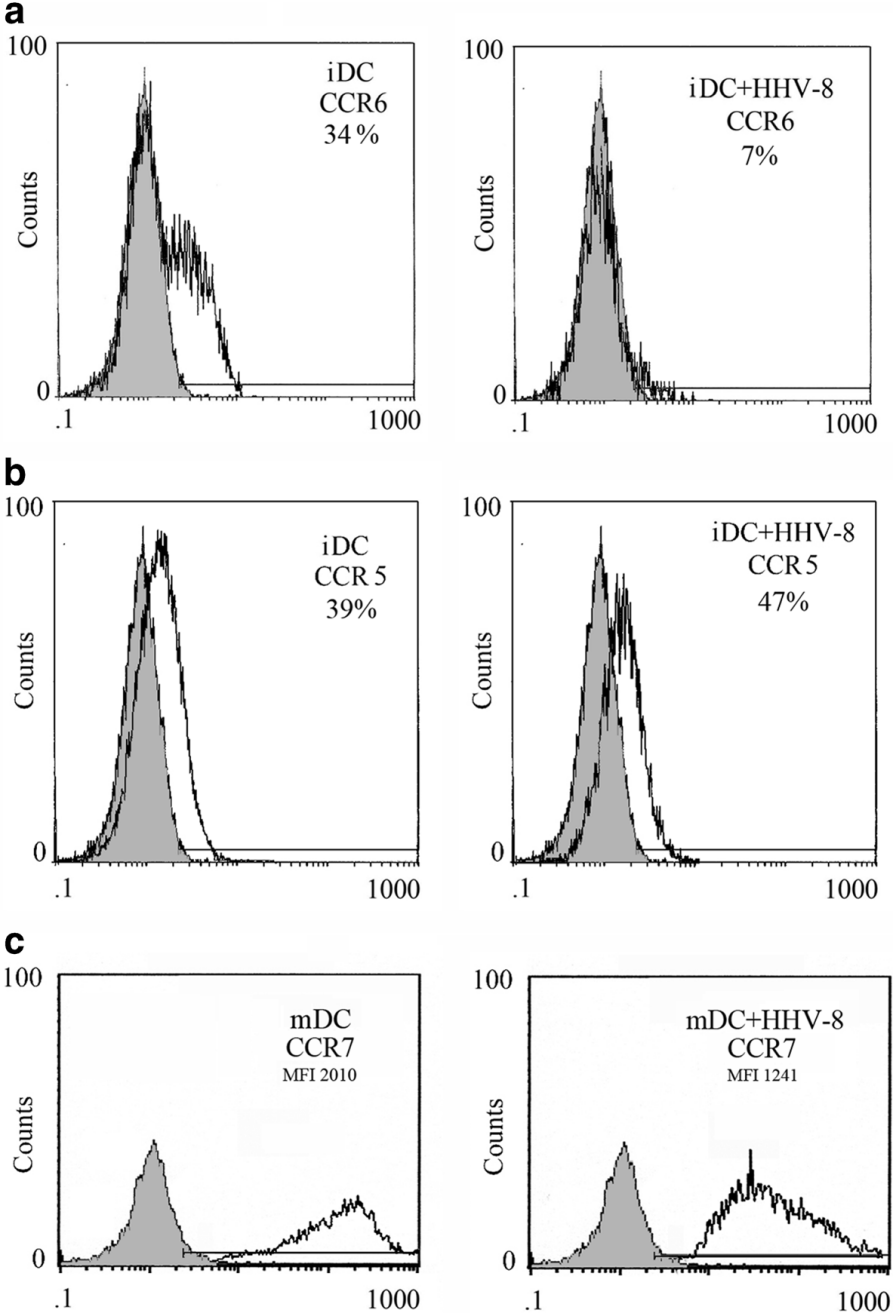
**Chemokine receptor expression on DCs exposed or not exposed to HHV-8**: flow cytometric analysis of chemokine receptor 5 and 6 (CCR5 and 6) expression on immature DCs obtained after six days of culture (**a** and **b**) and of chemokine receptor 7 (CCR7) expression on LPS-matured DCs (**c**), exposed or not exposed to HHV-8. One representative experiment out of three is shown. a) iDCs mock: % mean±SD = **27±9.9**, iDCs + HHV-8: % mean±SD =**8.5±2.2**; c) mDCs mock: MFI mean±SD = **2067±506**; mDC + HHV-8: MFI mean±SD = **1100±147.**

**Figure 2:**
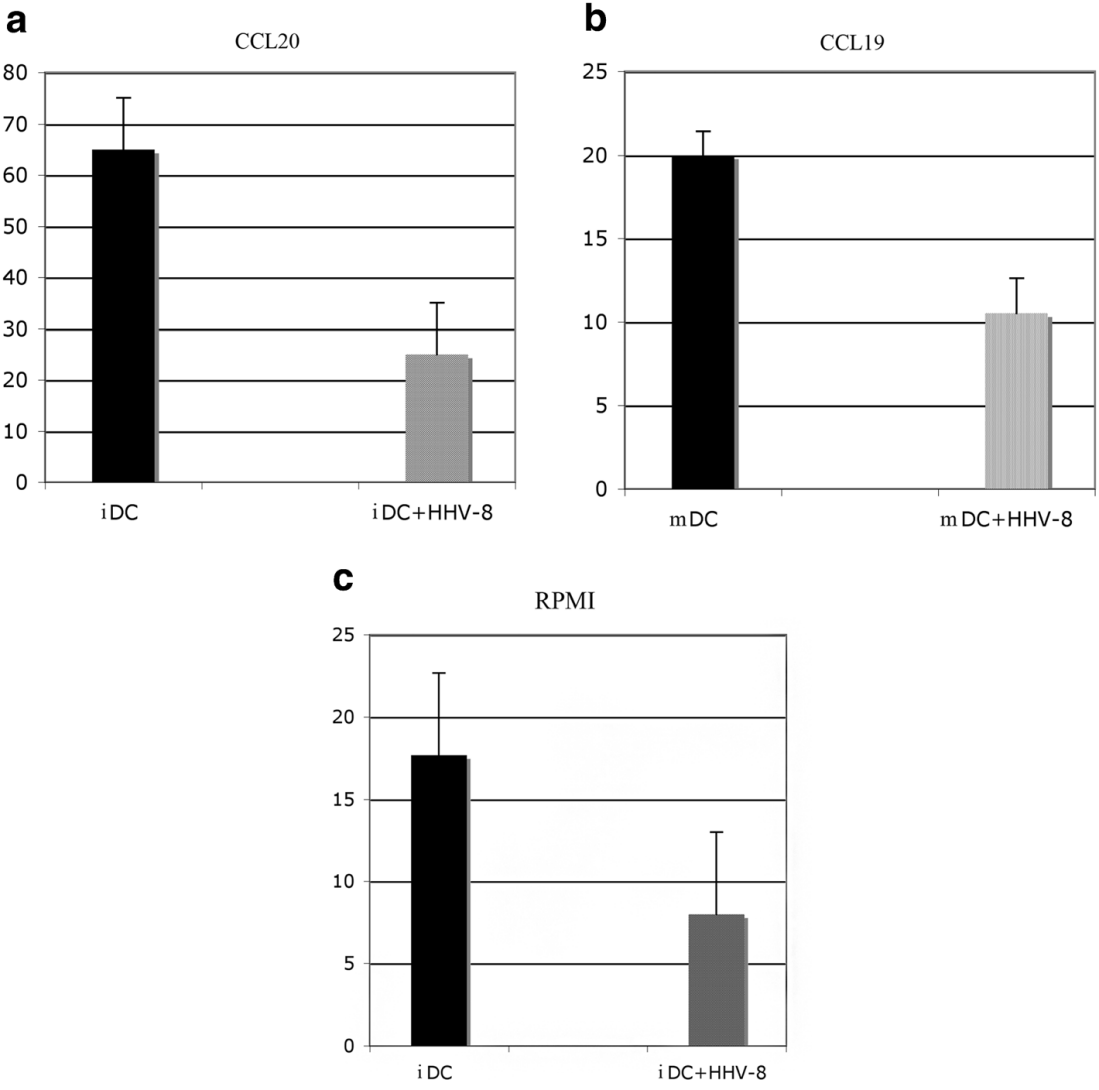
**DC migration assay**: immature and mature DCs exposed or not exposed to HHV-8 were allowed to migrate for 2 hours in response to CCL20 (**a**), CCL19 (**b**) or medium only (**c**). Mean plus SD of the percentage of initial imput of three independent experiments is indicated.

**Figure 3:**
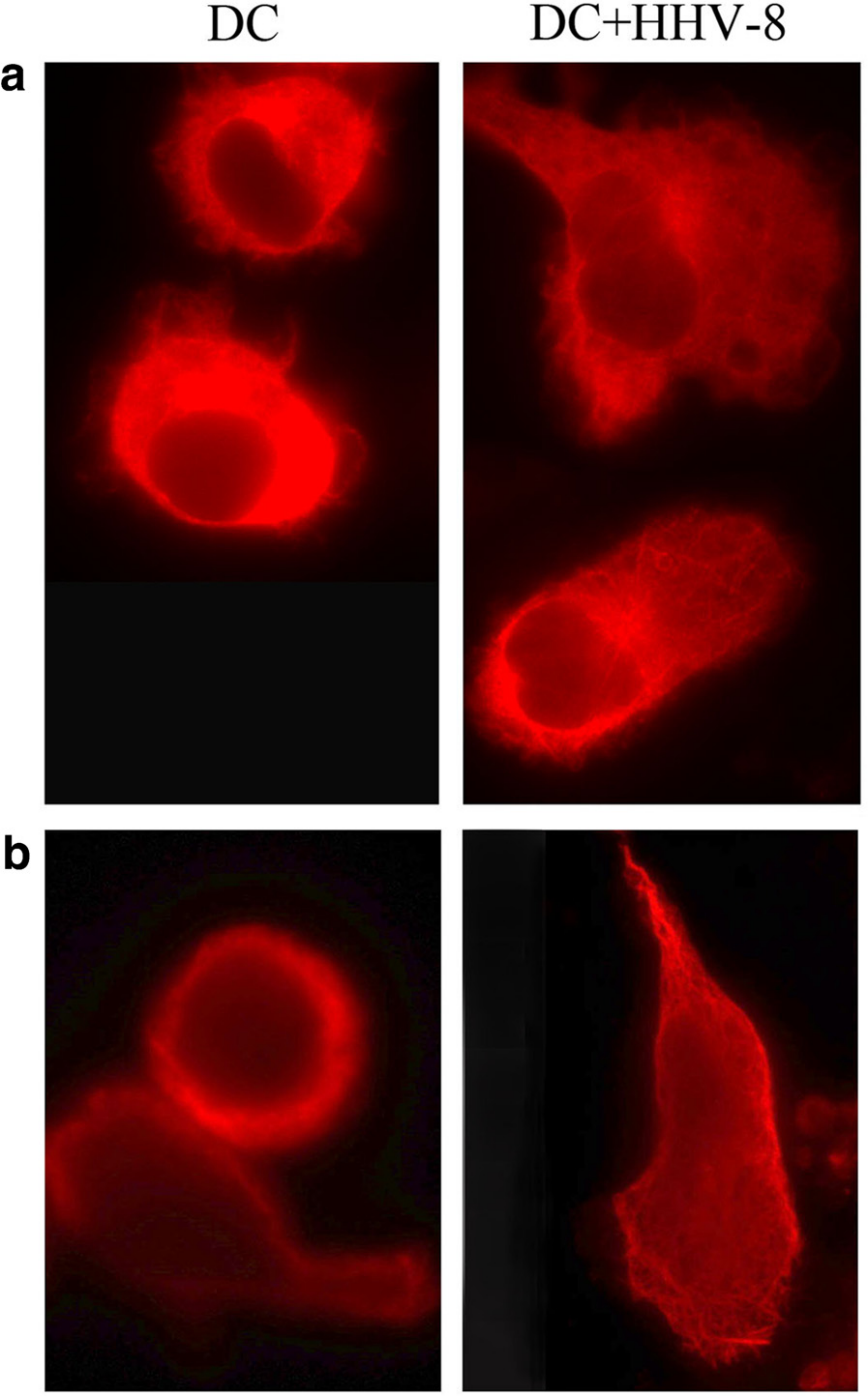
**HHV-8 infection induces cytoskeleton reorganization of DCs**: immunofluorescence analysis of fixed and permeabilized DCs, exposed or not exposed to HHV-8, using anti-tubulin (**a**) and anti-vimentin (**b**) monoclonal antibodies directed against the microtubules and intermediate filaments of the cytoskeleton.

CD14+ monocytes, obtained from healthy volunteers, were positively selected using anti-CD14 MAb-conjugated magnetic microbeads (Miltenyi Biotec). HHV-8 was obtained from the HHV-8 positive but EBV-negative PEL cell line BCBL-1 treated with 20 ng/ml tetradecanoyl phorbol acetate (TPA) for 5 days to induce HHV-8 replication. The virus stock was prepared as previously described [14]. HHV-8 infection was performed by incubating 1x10^6^ monocytes for 2 hours at 37 ° C with 100 μl of viral stock containing approximately 1x10^8^ –1x10^9^ viral copies/ml. Quantitation of viral yields was performed using polymerase chain reaction (PCR) amplification by comparison to the viral copy number of BCBL-1, which is known to have 70 viral copies/cell [15]. Purified monocytes, exposed or not to HHV-8, were cultured in RPMI 1640 containing 10 % heat inactivated fetal calf serum (Gibco) and recombinant human granulocyte-macrophage colony stimulating factor (GM-CSF) plus interleukin 4 (IL-4) (20 ng/ml each, Miltenyi-Biotec). After six days of infection, the viral antigen expression, evaluated by immunofluorescence, showed that approximately 5-10 % of iDCs were LANA-positive, whereas the viral lytic antigens were not expressed (data not shown), in accordance with our previous study [14]. At the same time the iDCs were analyzed by Flow Cytometry for the surface expression of CCR6 and CCR5. Following exposure to HHV-8 the iDCs showed a reduced expression of CCR6, in comparison to the mock-treated iDCs, while the CCR5 expression was not affected (Figure 1a and b). In contrast with a previous study [16] we found that CCR6 was expressed together with CCR5 on the surface of the monocyte-derived iDCs obtained in vitro from healthy donors. We then compared the exposed to HHV-8 or mock-treated DCs for their ability to acquire CCR7, after 24 hr treatment with LPS. We found that the DCs exposed to HHV-8 acquired CCR7 after LPS-exposure but the expression of CCR7 was lower, in terms of mean fluorescence intensity (MFI) (Figure 1c). It interesting to note that the reduction of chemokine receptor expression was substantial even if the percent of infected cells was low and this effect could be mediated by the viral binding to the DC surface molecules. To test if the altered chemokine receptor expression of DCs obtained after exposure to HHV-8 was accompanied by an altered chemotactic responsiveness to the corresponding chemokines, we performed a migration assay using the 96-transwell chemotaxis chambers containing 5 μm membrane inserts (Costar). 10^5^ iDCs, exposed or not exposed to HHV-8, counted by tripan blue exclusion, were allowed to migrate in response to CCL20, Rantes, MIP1 alfa and beta for 2 hours. The results obtained indicate that iDCs exposed to HHV-8 differ in the migration capacity towards CCL20 (Figure 2a), in agreement with the observed down-regulation of the corresponding CCR6 receptor, while the migration towards MIP1alfa, beta and Rantes was low and similar for both virus or mock treated DCs (data not shown). More importantly, the DCs exposed to HHV-8 and matured with LPS showed reduced migration towards CCL19, in comparison to the LPS-treated mock-infected DCs (Figure 2b). CCL19 is an important chemokine that drives mature DCs to the secondary lymphoid organs, where they can initiate the T cell response. The entire population of cells migrated to the lower wells were counted by flow cytometry. The migration assay was performed in duplicate with three different donors and the results are reported as means plus SD of the percentage of initial input. Finally, we observed that virus-exposed iDCs exhibited a reduced basal migration towards the medium without chemokines in comparison with the mock-treated iDCs (Figure 2c). The inhibition of spontaneous DCs motility induced by HHV-8 prompted us to investigate the organization of the cellular cytoskeleton. To this aim, we performed an immunofluorescence analysis of microtubules and intermediate filaments in DCs exposed or not exposed to HHV-8. The cells were fixed with 4 % paraformaldehyde in PBS for 30 min, washed in 0.1 M glycine, permeabilized in 0.1 % Triton X-100 and labeled with anti-vimentin and anti-tubulin monoclonal antibodies. As shown in Figure 3 we observed that the virus was responsible for reorganization of the microtubules (a) and vimentin (b) network that could contribute to the impairment of DCs motility.

**Figure 4:**
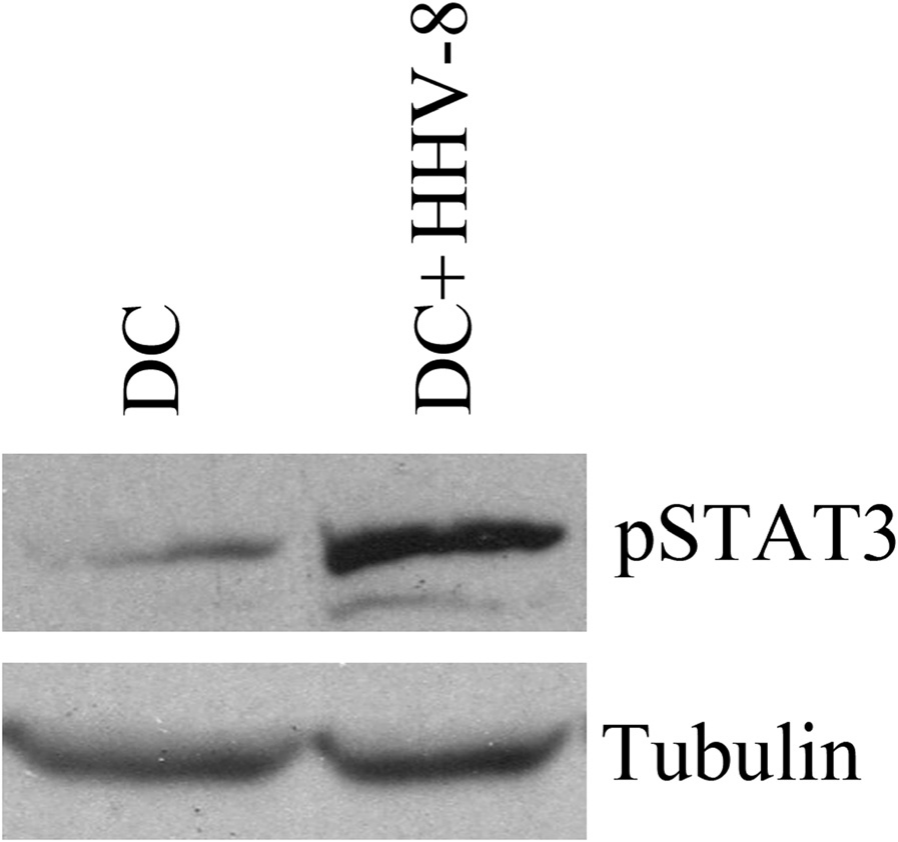
**HHV-8-induced STAT3 phosphorylation**: level of STAT3 phosphorylation (p-STAT3) in the iDCs mock and after HHV-8 exposure. Level of tubulin is also shown. Data for one of three representative donors are shown.

CCR1, 2 and 5 are essential for the recruitment of immature DCs to the peripheral tissues, where they initiate an effective immune response [4]. CCR6 seems to play a critical role in the recruitment of immature DCs and their precursors to sites of potential antigen-entry and in particular in the skin-homing of immature DCs and T cells [16]. Its specific interaction with CCL20 represents one of the exceptions to the general rule of promiscuity of chemokines and their receptors. After the antigen uptake and exposure to pro-inflammatory cytokines and factors, DCs mature and this process is accompanied by acquirement of chemokine receptors like CCR7 that drive the DCs to the secondary lymphoid organs [17]. Several viruses have developed different strategies to interfere with DC functions [18] and migration properties [7-9,11]. Among them HHV-8 has been previously shown by us and others to alter the DC cytokine production and reduce their ability to activate T cells [13,14,19]. However, the influence on the migratory ability of DCs, previously observed for other herpesviruses [9,10,20], has not yet been investigated for HHV-8. Our data, showing lower CCR6 expression on iDCs and CCR7 on LPS-matured DCs after exposure to HHV-8, together with the reduced migration towards the corresponding chemokines, indicate that the virus is able to interfere with several steps of DC traffic through the body. In a attempt to find an underlying molecular mechanism of DC dysfunction mediated by HHV-8, observed in this and our previous study [14], we investigated the signal trasducer and activator of transcription-3 (STAT3) phosphorylation status in DCs. STAT3 has been previously reported to be responsible for abnormal DC differentiation in other pathological situations [21]. We found that the iDCs, exposed to HHV-8 and differentiated in vitro for six days, showed hyperphosphorylation of STAT3 (Tyr-705) in comparison to the mock iDCs (Figure 4), suggesting that HHV-8 is able to activate this suppression pathway in DCs. We have also shown that HHV-8 induces cytoskeleton modifications impairing their spontaneous migration and reducing their basal motility. The present finding adds for the first time HHV-8 to the list of viruses that alter the migratory capacity of DCs as an immune escape mechanism.

## Competing interest

We have no competing financial or non financial interests.

## Authors’ contributions

MC designed the experiments; MC, VC, AF, SV, RS and MG performed the experiments; MC, PT, LF and AF wrote the manuscript. All authors read and approved the final version of the manuscript.
